# Exploring the threshold for the start of respiratory syncytial virus infection epidemic season using sentinel surveillance data in Japan

**DOI:** 10.3389/fpubh.2023.1062726

**Published:** 2023-02-03

**Authors:** Takeshi Miyama, Kensaku Kakimoto, Nobuhiro Iritani, Takayuki Nishio, Tomohiko Ukai, Yuka Satsuki, Yasutaka Yamanaka, Yoko Nishida, Ayumi Shintani, Kazushi Motomura

**Affiliations:** ^1^Epidemiology Section, Division of Public Health, Osaka Institute of Public Health, Osaka, Japan; ^2^Emergency Preparedness and Response Section, Division of Public Health, Osaka Institute of Public Health, Osaka, Japan; ^3^Department of Medical Statistics, Osaka Metropolitan University Graduate School of Medicine, Osaka, Japan

**Keywords:** epidemic threshold, early epidemic detection, effective reproduction number, epidemiology, Japan, prevention, relative operating characteristic curve analysis, respiratory syncytial virus (RSV)

## Abstract

**Introduction:**

An unusual seasonality of respiratory syncytial virus (RSV) infection in Japan is observed in recent years after 2017, becoming challenging to prepare for: a seasonal shift from autumn–winter to summer–autumn in 2017–2019, no major epidemic in 2020, and an unusually high number of cases reported in 2021.

**Methods:**

To early detect the start-timing of epidemic season, we explored the reference threshold for the start-timing of the epidemic period based on the number of cases per sentinel (CPS, a widely used indicator in Japanese surveillance system), using a relative operating characteristic curve analysis (with the epidemic period defined by effective reproduction number).

**Results:**

The reference values of Tokyo, Kanagawa, Osaka, and Aichi Prefectures were 0.41, 0.39, 0.42, and 0.24, respectively.

**Discussion:**

The reference CPS value could be a valuable indicator for detecting the RSV epidemic and may contribute to the planned introduction of monoclonal antibody against RSV to prevent severe outcomes.

## 1. Introduction

Respiratory syncytial virus (RSV) infection can cause bronchiolitis and pneumonia. Infants, the elderly, and individuals with immunodeficiency and/or congenital heart disease are considered at high risk of developing severe RSV infection (1). As of this writing, there is still no licensed vaccine against RSV, although one licensed humanized monoclonal antibody against RSV F glycoprotein, palivizumab (Synagis^Ⓡ^, AstraZeneca K.K., Osaka, Japan), is currently available. For high-risk infants and children, including preterm infants and those with bronchopulmonary dysplasia, congenital heart disease, immunodeficiency, or Down syndrome, the cost of palivizumab treatment to prevent the development of severe conditions is covered by the national health insurance in Japan (50 mg palivizumab = 55,000 yen). The dosage regimen of palivizumab is 15 mg/kg of body weight, and it is administered monthly during an anticipated RSV epidemic, which is consistent with the manufacturer's recommendations. Since October 2011, the use of the RSV antigen detection assay is covered for infant outpatients and outpatients for whom palivizumab is indicated; previously, only inpatients were covered (2). The detection of RSV epidemic season is important to ensure appropriate timing of palivizumab administration to prevent the occurrence of serious RSV infections in high-risk infants and children, because it is given monthly during the RSV season (3).

RSV is reported under a pediatric sentinel surveillance system in the National Epidemiological Surveillance of Infectious Diseases (NESID) Program in Japan. A pediatric sentinel site is selected per 30,000–50,000 population, and ~3,000 hospitals and clinics are registered as pediatric sentinel sites. The NESID returns the information on the weekly number of reported cases and reported cases per sentinel (CPS, average number of reported cases per sentinel) to the public; thus, the number of CPS is widely recognized and used in medical institutions in Japan to evaluate the level of incidence. Reference CPS value is used for the start of influenza epidemic season, and an announcement is released by the Ministry of Health, Labor and Welfare when the CPS exceeds the reference value (although, to our knowledge, the evidence to obtain this value is not publicly available). However, the reference CPS value for the start of an RSV epidemic has not been officially defined by the NESID program, and they do not announce an epidemic alert regarding RSV infection, making it difficult to prepare for an epidemic. Moreover, while RSV infections were more often observed in winter before 2017, a seasonal shift was observed to summer–autumn in Japan in 2017–2019 (4), and unusual RSV dynamics were observed during the coronavirus disease (COVID-19) pandemic (5–7) (no major epidemic in 2020, and an unusually high number of cases reported in 2021). Therefore, the timing of the announcement or prediction of an epidemic is even more difficult. Hence, to overcome the current situation and efficiently prepare for an epidemic, defining the reference CPS threshold could be useful.

A previous study investigated the reference CPS values for the start of the epidemic period in Japan (8) using RSV incidence data between 2012 and 2017, assuming that the annual epidemic seasonality follows the sinusoidal curve cycle (i.e., an annual regular cycle). Considering the irregular RSV dynamic patterns observed in recent years, the sinusoidal curve cycle assumption may not be applicable for the RSV incidence after 2017 in Japan. Contrarily, the effective reproduction number, *R*_*t*_, the average number of secondary cases produced by a single primary case at a specific time, *t*, in a given population (9), has been widely used to monitor trends in transmissions, regardless of the presence or absence of annual cyclicity. It has a threshold value of 1 to determine the phase of an epidemic. Monitoring *R*_*t*_ is valuable to evaluate the effects of interventions as seen in the COVID-19 pandemic (10, 11). It can also be used to evaluate the epidemic phase (whether it is in an ascending [i.e., *R*_*t*_ > 1] or decreasing [*R*_*t*_ < 1] phase) with high sensitivity even for the current RSV infection dynamics whose seasonality is irregular.

The relative operating characteristic (or receiver operating characteristic, ROC) curve analysis is used to determine the optimal threshold value of a diagnostic test that gives a dichotomous outcome (positive/negative test results) (12). For a test that reports the results on a continuous scale, the sensitivity and specificity can be calculated across all possible threshold values in comparison to gold standard status. The optimal threshold value is determined for the value where the sum of sensitivity and specificity is the maximum (see Youden index in Method section 2.3) (13). We considered that we can apply this method to detect the CPS threshold of RSV infection during epidemics. Each time datum of the CPS of RSV infection corresponds to the continuous scale test results above. We defined the epidemic period (assuming it is the true status of the epidemic period or gold standard) using *R*_*t*_ in the current study because *R*_*t*_ defines the ascending phase of the epidemic with high sensitivity (i.e., *R*_*t*_ can detect the start of epidemic seasons early).

The objective of this study is to determine the CPS threshold of the RSV infection epidemic season using ROC curve analysis. Since seasonality varies depending on the region (2) and region-dependent reference values are required (14), we explored region-dependent CPS thresholds of the epidemic season.

## 2. Materials and methods

### 2.1. Data

The weekly number of reported RSV infection cases and CPS were retrieved from the Infectious Diseases Weekly Report (15). The report is based on laboratory diagnosis using enzyme immunoassays to detect the RSV antigen and/or nucleic acid amplification tests for the corresponding RSV gene from nasal/throat swabs specimens. Ten prefectures whose population size was within the top 10 largest as of 2020 (Tokyo, Kanagawa, Osaka, Aichi, Saitama, Chiba, Hyogo, Hokkaido, Fukuoka, and Shizuoka) (16) were selected, and data between 2011 and 2019 were used. We selected these prefectures because the number of reported cases is expected to be large, which leads to a stable confidence interval of *R*_*t*_ (i.e., the interval is not too large). The study period was selected because of the considerable reporting rate change since October 2011 (2). The data used in this study does not include any personal information and they are publicly available; thus, ethical approval and consent are not required.

### 2.2. Epidemic period definition

**Figure 1:**
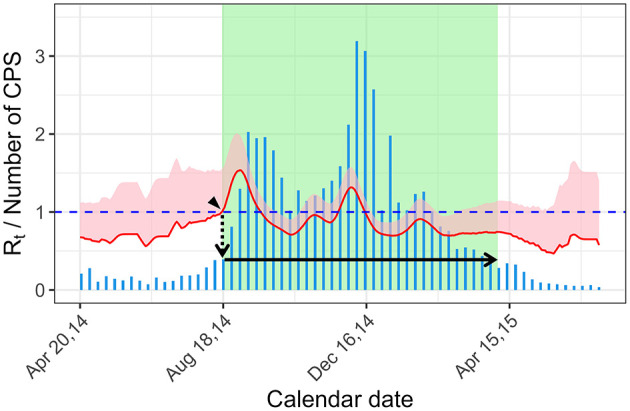
Definition of the epidemic period using the effective reproduction number. The start of the epidemic period is the time when the lower bound of the 95% CI became >1 (arrow head). The epidemic threshold of the year is the numbers of cases at the start of the epidemic period (dotted arrow). The end of the epidemic period is time when the number of cases is below the epidemic threshold (solid arrow). The bar graph (blue) shows the weekly CPS. The effective reproduction number is shown with 95% CI band (pink shade) and its lower bound (red line). Green rectangle shade shows the epidemic period. *R*_*t*_, effective reproduction number; CPS, case per sentinel.

In the current study, to determine the epidemic period of RSV infection as a true epidemic status (gold standard) for ROC curve analysis, we used the *R*_*t*_, which quantifies the potential for epidemic spread: if *R*_*t*_ is larger than 1, the infection would spread, whereas if *R*_*t*_ is < 1, the infection would die out. We define the start of the epidemic period as the time when the lower bound of 95% confidence interval (CI) is >1, considering its uncertainty (Figure 1, arrow head). Then, we obtained the number of cases at the start of the epidemic period (Figure 1, dotted arrow), and the end of epidemic period as the time when the number of cases became below that at the start of the epidemic (17, 18) (or that at the start of the epidemic of the next year in case the number of cases does not became below that at the start of the epidemic) (Figure 1, solid arrow). We assumed this epidemic period as the gold standard for an epidemic detection test that uses the number of CPS (see section 2.3 below).

*R*_*t*_ can be estimated by the ratio of the number of new infections generated at a specific time t, *I*(*t*), to the total infectiousness of the infected individuals at time t, $\overset{t}{\underset{s=1}{\sum }}I\left(t-s\right)\omega \left(s\right)$, as below


$$\begin{array}{l} R_{t}=\frac{I(t)}{\sum_{s=1}^{t}I(t-s)\omega (s)} \end{array}$$

where ω(.) is the probability function of an infectivity profile (9). *R*_*t*_ was calculated using an R package, EpiEstim (9), over a 1-week time window. For the infectivity profile, ω(.), a serial interval (the time between the symptom onset of a primary case and that of secondary case) was used. The mean and standard deviation of the serial interval for RSV infection was assumed to be 7.5 and 2.1 days, respectively (19). Since the above *R*_*t*_ estimation uses *I*(.) and ω(.) which are on a daily scale, data on the daily number of cases are required. We derived the daily data of RSV cases by fitting the cumulative number of weekly data to smoothing spline (20, 21). Then, the daily incidence was obtained by monitoring the daily difference of the estimated smoothing spline. The smoothing spline was estimated by the function “smooth.spline” in splines package in R (22) to minimize the penalized least squares using the generalized cross-validation criterion. To make the estimated values as count data, they were rounded to the nearest integer. When the calculated number of daily cases was zero, it was replaced with 1 to avoid an extreme fluctuation of *R*_*t*_ estimation when the number of cases is small.

### 2.3. Exploring the reference value for the start of epidemic using the number of reported cases per sentinel

Since the CPS in the NESID system in Japan has been widely used, rather than *R*_*t*_, among health care workers and medical institutions, the reference value based on CPS to detect the start of the epidemic period could be useful (2, 8). Evaluating epidemic periods using the CPS threshold (CPS method) is relatively simple: it is positive (i.e., in an epidemic period) if the CPS at a certain period is larger or equal to the threshold value, and it is negative (i.e., not in an epidemic period) if the CPS at a certain period is smaller than the threshold. Now, we need to identify the cut-off value of the CPS that could detect the epidemic period defined in section 2.2 (the gold standard) with high sensitivity and specificity for. To do so, we performed an ROC curve analysis and plot an ROC curve by calculating the sensitivity and specificity against the gold standard for candidate CPS cut-off values of the CPS method. We determined the most plausible cut-off value as the reference CPS value for the start of epidemic, maximizing the Youden index (13). Youden index is calculated as follows:


$$\begin{array}{l} \text{Youden}\;\text{index}=\text{sensitivity}+\text{specificity}-1. \end{array}$$

The reference values were calculated for each prefecture using the incidence data in 2012–2019, 2012–2015, and 2016–2019 to show period-dependency.

## 3. Results

### 3.1. Epidemic period defined by the effective reproduction number

**Figure 2:**
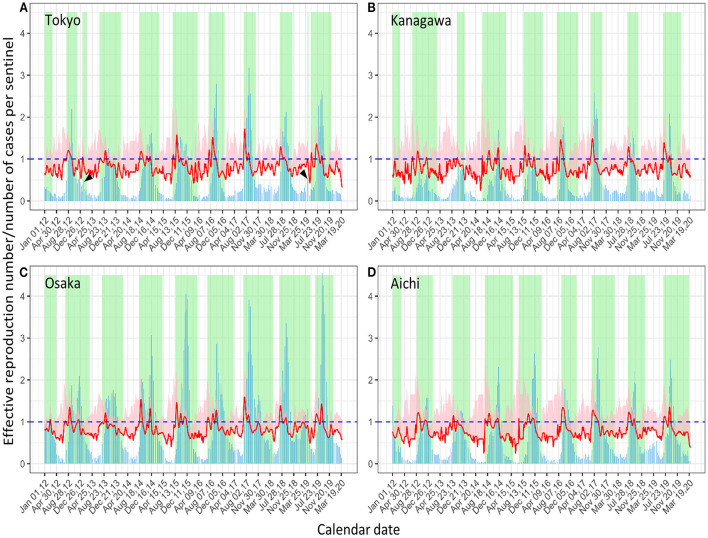
Number of cases per sentinel (CPS) of respiratory syncytial virus infection from 2012 to 2019 with the effective reproduction number. **(A–D)** Tokyo **(A)**, Kanagawa **(B)**, Osaka **(C)**, and Aichi **(D)** Prefectures. The bar graph (blue) shows weekly CPS. The effective reproduction number is shown with 95% CI band (pink shade) and its lower bound (red line). The green rectangle shade shows the epidemic period (please refer to the main text and Figure 1 for the definition of the epidemic period). By this definition, small surges may be included (arrow heads).

The epidemic curves of Tokyo, Kanagawa, Osaka, and Aichi are shown in Figure 2 with *R*_*t*_ and defined epidemic periods. The epidemic periods, which are defined using *R*_*t*_ as the gold standard for the ROC curve analysis (Methods, section 2.2), are shown in green. The epidemic periods captured the waves of the epidemic curves where the CPS values were high in general (e.g., Figure 2C in Osaka). In some occasions, short periods with a small surge before and after the main waves (e.g., Figure 2A, head arrows in 2012 and 2019) were defined as epidemics. The epidemic curve, *R*_*t*_, and epidemic periods of the other prefectures are shown in Supplementary Figure 1.

### 3.2. Reference value for the start of the epidemic period by the number of reported cases per sentinel

From the results of the ROC curve analysis, the reference CPS values for the prefectures whose population size is within the top 10 largest in Japan in 2012–2019, 2012–2015, and 2016–2019 ranged from 0.26 to 0.74, from 0.16 to 0.97, and from 0.24 to 0.81, respectively (Table 1). The reference values of the prefectures whose population size is within the top 4 largest (Tokyo, Kanagawa, Osaka, and Aichi) in 2016–2019 were 0.41, 0.39, 0.42, and 0.24, respectively. The reference values varied among prefectures. For example, in 2012–2019, the reference values in the studied prefectures, except Hokkaido and Fukuoka, were between ~0.3 and 0.5, whereas Hokkaido and Fukuoka had the higher reference value between 0.6 and 0.7, as compared to the other prefectures. The reference values obtained from 2016 to 2019 were generally higher than those from 2012–2015 (except for Aichi, Hyogo, and Hokkaido).

**Table 1:** Reference values using the number of cases per sentinel for the start of the epidemic season.

**Prefecture**	**Period**	**Reference value** **(case/sentinel)**	**Sensitivity**	**Specificity**	**Youden index**	**AUC (95% confidence interval)**
Tokyo	2012–2019	0.30	0.84	0.80	0.64	0.87 (0.85–0.88)
	2012–2015	0.21	0.89	0.90	0.79	0.93 (0.92–0.94)
	2016–2019	0.41	0.85	0.87	0.72	0.88 (0.86-0.90)
Kanagawa	2012–2019	0.32	0.66	0.88	0.54	0.82 (0.81–0.84)
	2012–2015	0.18	0.86	0.82	0.67	0.89 (0.88–0.91)
	2016–2019	0.39	0.69	0.88	0.57	0.80 (0.77–0.82)
Osaka	2012–2019	0.39	0.91	0.98	0.89	0.98 (0.98–0.99)
	2012–2015	0.35	0.93	0.95	0.87	0.98 (0.98–0.99)
	2016–2019	0.42	0.91	0.98	0.89	0.98 (0.98–0.99)
Aichi	2012–2019	0.28	0.79	0.90	0.69	0.90 (0.89–0.91)
	2012–2015	0.31	0.72	0.99	0.71	0.88 (0.86–0.90)
	2016–2019	0.24	0.93	0.79	0.72	0.94 (0.93–0.95)
Saitama	2012–2019	0.48	0.72	0.88	0.60	0.86 (0.85–0.87)
	2012–2015	0.16	0.89	0.58	0.47	0.80 (0.78–0.82)
	2016–2019	0.48	0.79	0.97	0.76	0.94 (0.93–0.96)
Chiba	2012–2019	0.30	0.74	0.94	0.68	0.85 (0.83–0.86)
	2012–2015	0.30	0.66	0.97	0.63	0.84 (0.82–0.86)
	2016–2019	0.33	0.84	0.94	0.78	0.92 (0.90–0.94)
Hyogo	2012–2019	0.26	0.83	0.61	0.43	0.75 (0.73–0.76)
	2012–2015	0.97	0.41	0.85	0.26	0.65 (0.62–0.68)
	2016–2019	0.29	0.87	1.00	0.87	0.96 (0.95–0.97)
Hokkaido	2012–2019	0.74	0.93	0.73	0.66	0.88 (0.87–0.89)
	2012–2015	0.76	0.96	0.88	0.84	0.96 (0.95–0.97)
	2016–2019	0.74	0.90	0.59	0.48	0.78 (0.76–0.80)
Fukuoka	2012–2019	0.64	0.80	0.70	0.51	0.81 (0.79–0.82)
	2012–2015	0.42	0.92	0.97	0.90	0.98 (0.97–0.99)
	2016–2019	0.81	0.81	0.74	0.55	0.80 (0.77–0.82)
Shizuoka	2012–2019	0.38	0.70	0.72	0.42	0.72 (0.70–0.74)
	2012–2015	0.34	0.74	1.00	0.74	0.90 (0.88–0.91)
	2016–2019	0.69	0.57	0.73	0.30	0.62 (0.58–0.67)

Sensitivity, specificity, Youden index, and AUC (area under the ROC curve) were calculated from the relative operating characteristic curve analyses.

The ROC curves for the prefecture- and period-dependent analyses are shown in Supplementary Figures 2–4.

## 4. Discussion

We conducted an ROC curve analysis to determine the reference value of CPS that plausibly determines the start of an RSV epidemic, which is required for a planned administration of palivizumab to high-risk infants and the elderly. From the current study, we found that the reference values for prefectures whose population size is within the top 10 largest in Japan were all < 1, showing ranges of 0.26–0.74, 0.16–0.97, and 0.24–0.81in 2012–2019, 2012–2015, and 2016–2019, respectively.

Another investigation on the start of the epidemic period in Japan (8) showed similar result as our study using the RSV incidence data between 2012 and 2017, reporting that the reference CPS value ranged from 0.26 to 0.61 for the 10 prefectures. The previous study used the sinusoidal curve cycle to capture the RSV epidemic seasonality, which may not be able to capture the recent RSV seasonality change (4, 8), especially during the COVID-19 pandemic period (5–7). However, the method to detect the start of the epidemic using the effective reproduction number, which was applied in the current study, is not affected by the cyclicity change. Moreover, this may be applicable for other infectious diseases with annual seasonality or other surveillance systems used in other countries. Another method to determine epidemic periods is the moving epidemic method (MEM), which is used for infectious diseases such as influenza (23) and RSV (24) and is not influenced by seasonality. The MEM algorithm identifies the epidemic periods where the number of cases is high, while *R*_*t*_ identifies the increase (change) in the number of cases. The characteristics of these two methods are different, and further research is needed to evaluate how they influence the reference threshold.

The reference CPS values for the start of the epidemic period were not consistent among prefectures. The reference values of Hokkaido and Fukuoka in 2012–2019 were 0.6 and 0.7, respectively, which were higher than those of the other prefectures (0.3–0.5). This was because the incidence at baseline (non-epidemic period) was relatively high in Hokkaido and Fukuoka (i.e., the number of CPS in the non-green-shaded periods in Supplementary Figures 1D, E was higher than the other regions). If the baseline incidence level is high, the incidence level when the *R*_*t*_ becomes >1 will consistently be high, since the reproduction number is the ratio between the number of primary and secondary cases. As the reference CPS values in this study varied among prefectures and the epidemic season in each region is slightly different, a regional-level reference CPS value should be obtained when applying our method (if the national-level CPS threshold is obtained, the sensitivity of the CPS method could be lower). Moreover, the reference CPS value for the start of the epidemic period varied depending on the time. The reference values in 2016–2019 were in general slightly higher than those in 2012–2015. This is because the baseline incidence level has gradually increased from 2012 to 2019. The baseline incidence might be influenced by factors such as the reporting rate, scale of RSV infection sentinel sites selected in each prefecture, and balance of the number of susceptible and transmissibility of the disease. The specific reason for the region- or time-dependency of the incidence level at baseline is unclear; however, annual or 2–3-year updates by region could be a method to deal with the issue of time-dependency except in cases wherein an irregular season is observed such as during the COVID pandemic.

This study includes several limitations. First, it is difficult to define this epidemic period using the lower bound of *R*_*t*_ 95% CI for regions where the number of reported cases is small, because the 95% CI of *R*_*t*_ estimation would be too large. In the 13 prefectures (among the 47 prefectures in Japan), which were not included in the current study area, all epidemic periods could not be detected using this method (data are not shown). However, the target population of the current study (the 10 prefectures) did not have such a problem during the epidemic period estimation; thus, the CPS reference values for the start of the epidemic period estimated by the current study should be reasonable. A larger-level region may be used to estimate *R*_*t*_ for prefectures with a small number of reported cases. Second, the CPS threshold that was obtained in this study does not capture the epidemic period perfectly. Whether the sensitivity and specificity of the CPS reference values are acceptable depends on the users' context. However, we consider that they are an important metric as the index of CPS is easy to understand and commonly used to announce the current situation of RSV dynamics.

In conclusion, we estimated the CPS reference values that indicate the start of an RSV epidemic for prefectures whose population size is within the top 10 largest, which ranged from 0.16 to 0.97 in 2012–2015, and from 0.24 to 0.81 in 2016–2019 (e.g., Tokyo, 0.41; Kanagawa, 0.39; Osaka, 0.42 in 2016–2019). The reference CPS values should be estimated by regions and periods, considering their variations, and should be updated regularly. The reference CPS value can be a valuable indicator to detect an RSV infection epidemic season at the early stage, and may contribute to the planned administration of palivizumab, a monoclonal antibody against RSV, to prevent the severe outcome of RSV infections.

## Data availability statement

Publicly available datasets were analyzed in this study. This data can be found here: https://www.niid.go.jp/niid/en/idwr-e.html.

## Ethics statement

Ethical review and approval was not required for the study on human participants in accordance with the local legislation and institutional requirements. Written informed consent for participation was not required for this study in accordance with the national legislation and the institutional requirements.

## Author contributions

TM, NI, TU, KK, and KM conceptualized the study design. TM, TN, YN, YS, and YY collected the data. TM and AS analyzed the data. TM drafted the manuscript. All authors gave comments on the earlier versions of the manuscript, edited the manuscript, and approved the final version.

## References

[1] Centers for Disease Control Prevention. People at High Risk for Severe RSV Infection. (2022). Available online at: https://www.cdc.gov/rsv/high-risk/index.html (accessed August 30, 2022).

[2] National National Institute of Infectious Diseases Tuberculosis Tuberculosis and Infectious Diseases Control Division Ministry of Health Labour and Welfare. Respiratory syncytial virus infection. Infect Agents Surveill Rep. (2018) 39:207–9.

[3] World Health Organization. WHO Preferred Product Characteristics of Monoclonal Antibodies for Passive Immunization Against Respiratory Syncytial Virus (RSV) Disease. Geneva: World Health Organization. (2021).

[4] Miyama T Iritani N Nishio T Ukai T Satsuki Y Miyata H. Seasonal shift in epidemics of respiratory syncytial virus infection in Japan. Epidemiol Infect. (2021) 149:e55. 10.1017/S0950268821000340 33568242 PMC8060823

[5] Satsuki Y Motomura K Nishida Y Kakimoto K Nishio T Miyama T. Surveillance of infectious diseases in 2020 in Osaka Prefecture (in Japanese). Annu Rep Osaka Inst Public Health. (2021) 5:1−10.

[6] Ujiie M Tsuzuki S Nakamoto T Iwamoto N Ujiie M. Resurgence of respiratory syncytial virus infections during COVID-19 pandemic, Tokyo, Japan. Emerg Infect Dis. (2021) 27:2969–70. 10.3201/eid2711.211565 34388086 PMC8544984

[7] Yamanaka Y Satsuki Y Nishida Y Kakimoto K Ukai T Nishio T Miyama T Iritani N Motomura K. Surveillance of infectious diseases in Osaka Prefecture in 2021. Annu Rep Osaka Inst Public Health. (in press) 6.

[8] Yamagami H Kimura H Hashimoto T Kusakawa I Kusuda S. Detection of the onset of the epidemic period of respiratory syncytial virus infection in Japan. Front public Heal. (2019) 7:39. 10.3389/fpubh.2019.00039 30931290 PMC6425940

[9] Cori A Ferguson NM Fraser C Cauchemez S. A new framework and software to estimate time-varying reproduction numbers during epidemics. Am J Epidemiol. (2013) 178:1505–12. 10.1093/aje/kwt133 24043437 PMC3816335

[10] Nakajo K Nishiura H. Assessing interventions against coronavirus disease 2019 (COVID-19) in Osaka, Japan: a modeling study. J Clin Med. (2021) 10:1256. 10.3390/jcm10061256 33803634 PMC8003080

[11] Pan A Liu L Wang C Guo H Hao X Wang Q. Association of public health interventions with the epidemiology of the COVID-19 outbreak in Wuhan, China. JAMA. (2020) 323:1915–23. 10.1001/jama.2020.6130 32275295 PMC7149375

[12] Hajian-Tilaki K. Receiver operating characteristic (ROC) curve analysis for medical diagnostic test evaluation. Casp J Intern Med. (2013) 4:627–35. 24009950 PMC3755824

[13] Youden WJ. Index for rating diagnostic tests. Cancer. (1950) 3:32–35. 15405679 10.1002/1097-0142(1950)3:1<32::aid-cncr2820030106>3.0.co;2-3

[14] Center for Surveillance Immunization and Epidemiologic Research. RSV infection surveillance: historic trends and future consideration (in Japanese). Infect Agents Surveill Rep. (2018) 39:210–1.

[15] National Institute of Infectious Diseases. Infectious Diseases Weekly Report (IDWR). (2020). Available online at: https://www.niid.go.jp/niid/en/idwr-e.html (accessed January 6, 2020).

[16] Statistics Bureau Ministry of Internal Affairs and Communications Japan. “Population and Households,” in Japan Statistical Yearbook 2022. Tokyo: Statistics Bureau Ministry of Internal Affairs and Communications Japan. (2021). p. 8–32.

[17] WHO Regional Office for Europe U.S. CDC. WHO Regional Office for Europe Guidance for Sentinel Influenza Surveillance in Humans. Copenhagen: WHO Regional Office for Europe. (2011).

[18] Pan American Health Organization WHO Regional Office for the Americas. Operational Guidelines for Sentinel Severe Acute Respiratory Infection (SARI) Surveillance. (2014). Available online at: https://www3.paho.org/revelac-i/wp-content/uploads/2015/10/2015-cha-operational-guidelines-sentinel-sari.pdf (accessed July 12, 2022).

[19] Vink MA Bootsma MCJ Wallinga J. Serial intervals of respiratory infectious diseases: a systematic review and analysis. Am J Epidemiol. (2014) 180:865–75. 10.1093/aje/kwu209 25294601

[20] Nishiura H Chowell G. Early transmission dynamics of Ebola virus disease (ECD), West Africa, March to August 2014. Eurosurveillance. (2014) 19:1–6. 10.2807/1560-7917.ES2014.19.36.20894 25232919

[21] Yamauchi T Takeuchi S Yamano Y Kuroda Y Nakadate T. Estimation of the effective reproduction number of influenza based on weekly reports in Miyazaki Prefecture. Sci Rep. (2019) 9:1–9. 10.1038/s41598-019-39057-w 30796315 PMC6384943

[22] Wang W Yan J. Shape-restricted regression splines with R package splines2. J Data Sci. (2021) 19:498–517. 10.6339/21-JDS1020

[23] Vega T Lozano JE Meerhoff T Snacken R Mott J Ortiz de Lejarazu R Nunes B. Influenza surveillance in Europe: establishing epidemic thresholds by the moving epidemic method. Influenza Other Respi. Viruses. (2013) 7:546–58. 10.1111/j.1750-2659.2012.00422.x 22897919 PMC5855152

[24] Vos LM Teirlinck AC Lozano JE Vega T Donker GA Hoepelman AI. Use of the moving epidemic method (MEM) to assess national surveillance data for respiratory syncytial virus (RSV) in the Netherlands, 2005 to 2017. Euro Surveill. (2019) 24:1800469. 10.2807/1560-7917.ES.2019.24.20.1800469 31115311 PMC6530251

