# Supplementary material for: Exploring the threshold for the start of respiratory syncytial virus infection epidemic season using sentinel surveillance data in Japan
Source: Front Public Health. 2023 Feb 3;11:1062726. doi: 10.3389/fpubh.2023.1062726 (PMC9936060; doi:10.3389/fpubh.2023.1062726)
Supplement: Supplementary file 1 [file Data_Sheet_1.docx]

Supplementary Material

# Supplementary Figures


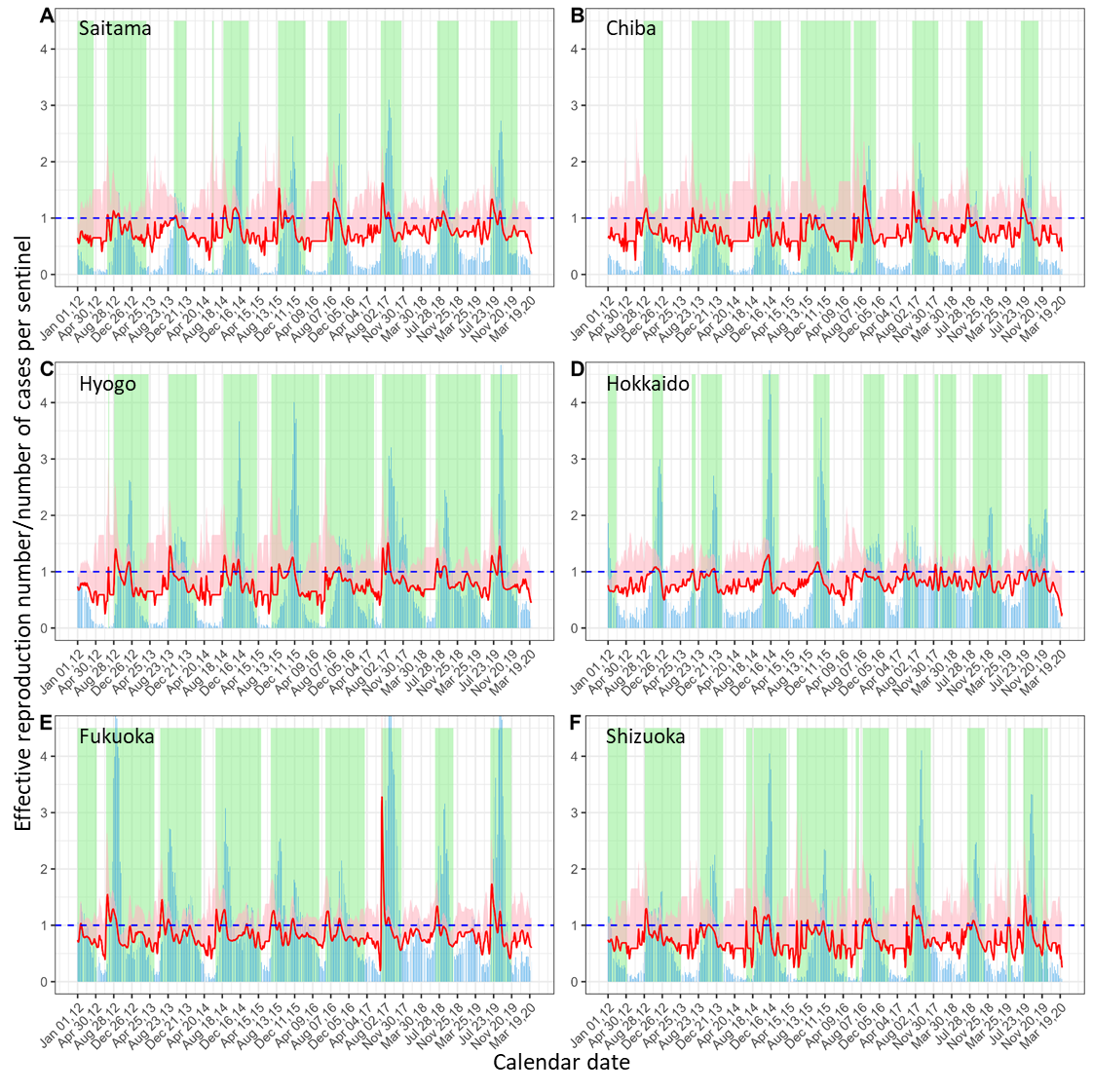


**Supplementary figure 1. The number of cases per sentinel (CPS) of respiratory syncytial virus infection from 2021 to 2019 with the effective reproduction number.** A–F) Saitama (A), Chiba (B), Hyogo (C), Hokkaido (D), Fukuoka (E), and Shizuoka (F) Prefectures. The bar graph (blue) shows weekly CPS. The effective reproduction number is shown with 95% confidence interval (pink shade) and its lower bound (red line). The green shade shows the epidemic period (please refer to the main text for the definition of the epidemic period).


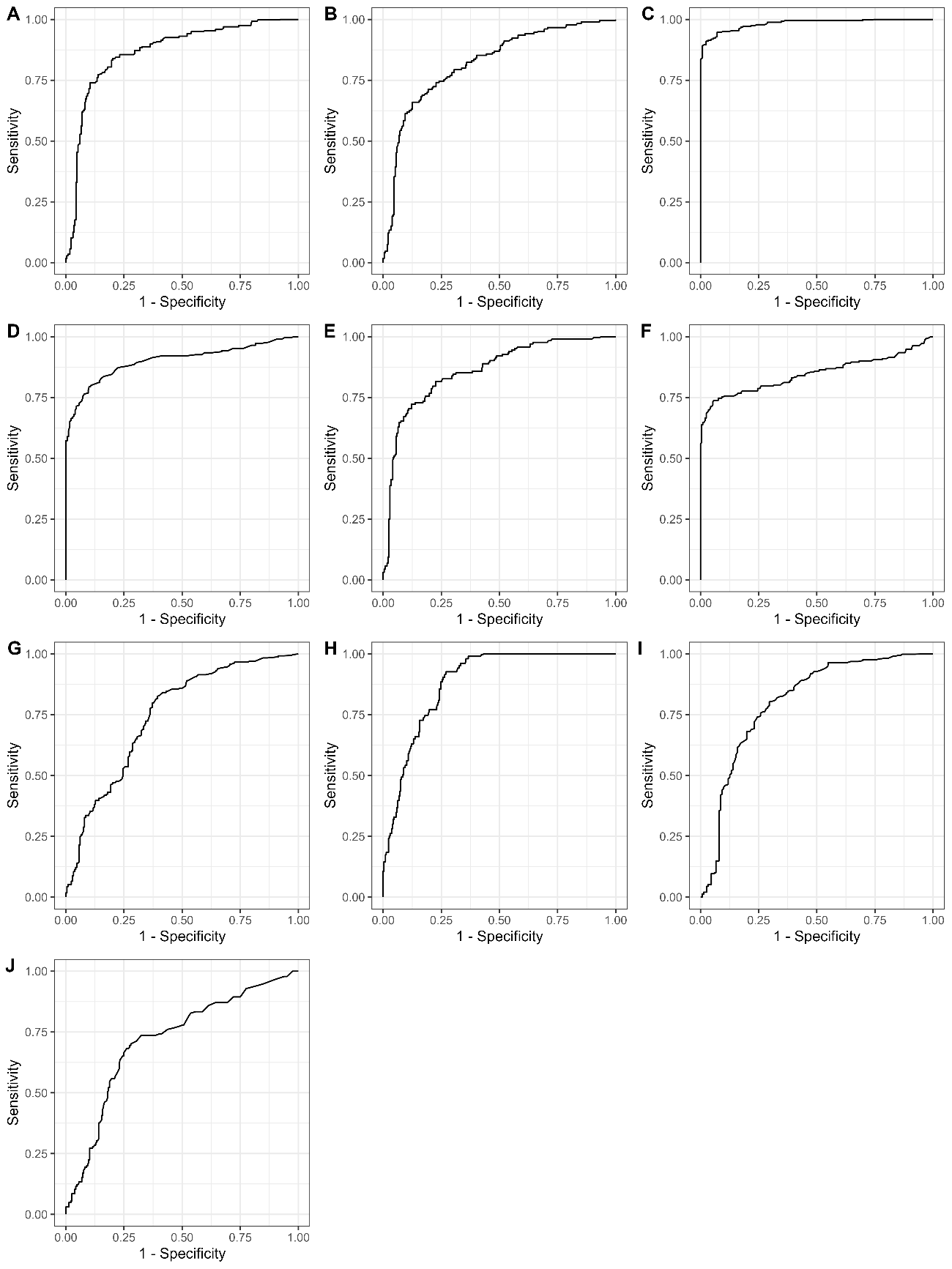


**Supplementary figure 2. Relative operating characteristic (ROC) curve using the cutoff values of the number of cases per sentinel (CPS) against the epidemic period between 2012 and 2019.** Please refer to the main text for the definition of the epidemic period. A–J: ROC curves for Tokyo (A), Kanagawa (B), Osaka (C), Aichi (D), Saitama (E), Chiba (F), Hyogo (G), Hokkaido (H), Fukuoka (I), and Shizuoka (J) Prefectures.

**
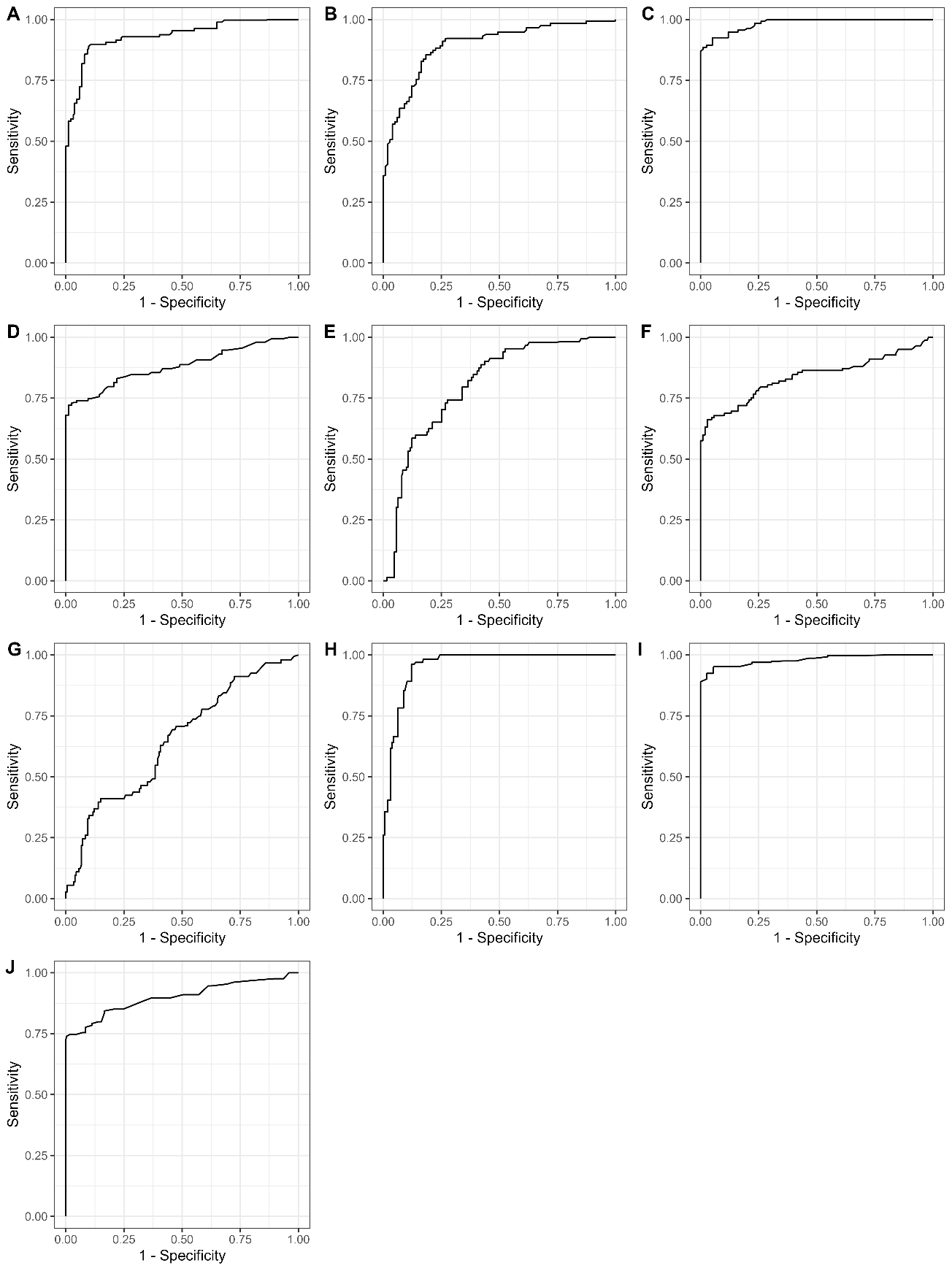
 Supplementary figure 3. Relative operating characteristic (ROC) curve using the cutoff values of the number of cases per sentinel (CPS) against the epidemic period between 2012 and 2015.** Please refer to the main text for the definition of the epidemic period. A–J: ROC curves for Tokyo (A), Kanagawa (B), Osaka (C), Aichi (D), Saitama (E), Chiba (F), Hyogo (G), Hokkaido (H), Fukuoka (I), and Shizuoka (J).

**
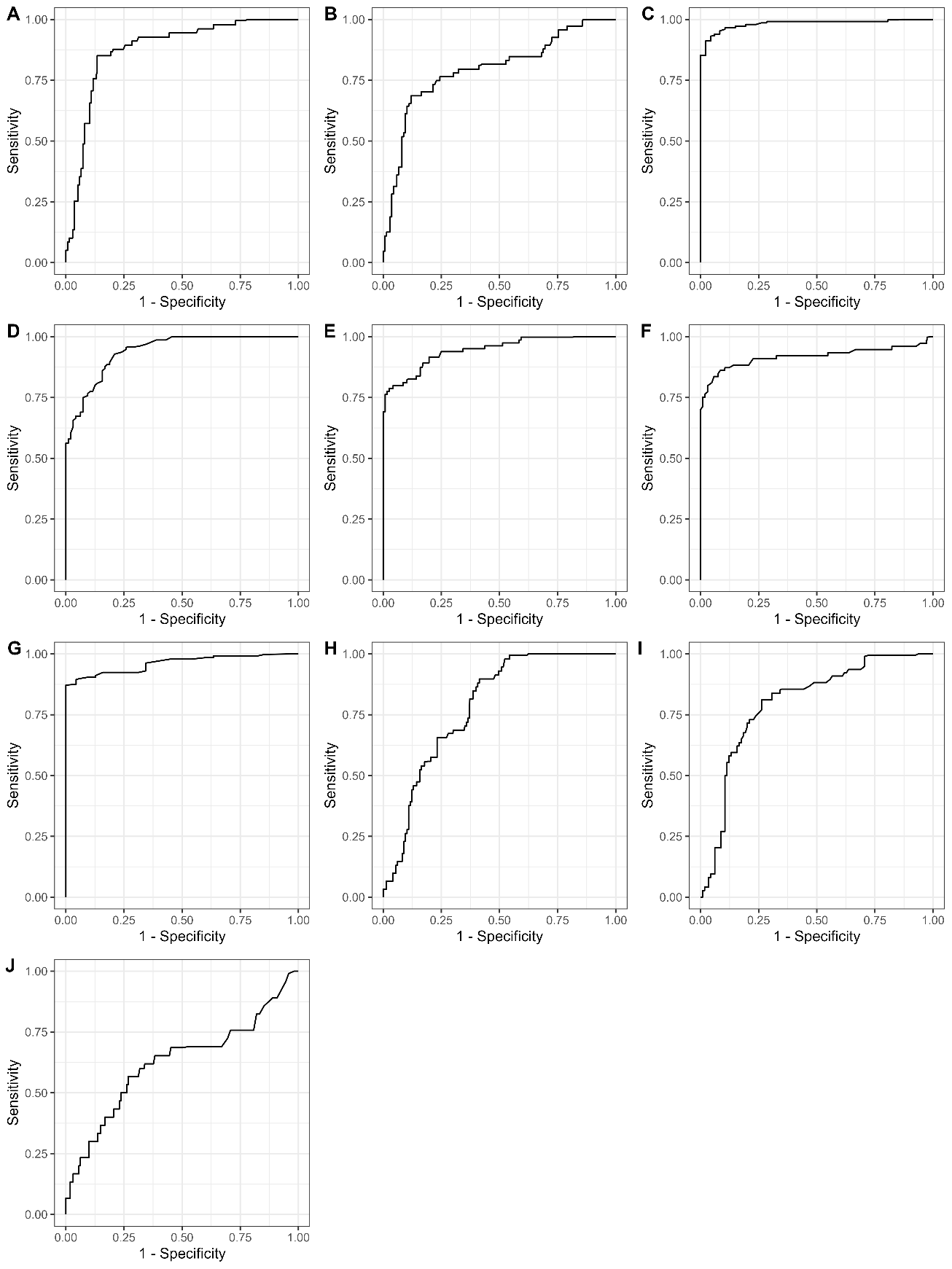
Supplementary figure 4. Relative operating characteristic (ROC) curve using the cutoff values of the number of cases per sentinel (CPS) against the epidemic period between 2016 and 2019.** Please refer to the main text for the definition of the epidemic period. A–J: ROC curves for Tokyo (A), Kanagawa (B), Osaka (C), Aichi (D), Saitama (E), Chiba (F), Hyogo (G), Hokkaido (H), Fukuoka (I), and Shizuoka (J).
